# A scoping review of medical education research for residents in radiation oncology

**DOI:** 10.1186/s12909-020-1927-x

**Published:** 2020-01-13

**Authors:** Ching-Hsin Lee, Po-Jui Chen, Hung-Yi Lai, Ching-Yi Lee, Kang-Hsing Fan, Ngan-Ming Tsang, Joseph Tung-Chieh Chang

**Affiliations:** 1 Department of Radiation Oncology, Proton and Radiation Therapy Center, Chang Gung Memorial Hospital at Linkou, No.5, Fuxing St., Guishan Dist, Taoyuan City, 333 Taiwan; 2 Chang Gung Medical Education Research Centre (CG-MERC), Chang Gung Memorial Hospital at Linkou, No.5, Fuxing St., Guishan Dist, Taoyuan City, 333 Taiwan; 3 Department of Neurosurgery, Chang Gung Memorial Hospital at Linkou, No.5, Fuxing St., Guishan Dist, Taoyuan City, 333 Taiwan

**Keywords:** Medical education, Resident, Scoping review, Radiation oncology

## Abstract

**Background:**

Both medical education and radiation oncology have progressed significantly in the past decade, but a generalized overview of educational research for radiation oncology residents has not been produced. This study examines recent research trends in medical education for residents in radiation oncology through a scoping review.

**Methods:**

We conducted a scoping review of medical education research for residents in radiation oncology to survey the research trends. We used publications available on MEDLINE, PubMed, and Scopus to conduct this scoping review.

**Results:**

We screened 221 full-text articles, 146 of which met our inclusion criteria. These publications showed increased activity in medical education research for residents, most involving affiliations in the United States. We identified persistent interest in training-, contouring-, and technology-related issues. An increase in research related to career, treatment quality, and multidisciplinary training was also observed. However, no research about teacher training was identified.

**Conclusions:**

This scoping review presents the trends in study interests among stakeholders of medical education research in radiation oncology. With an investigation of existing studies, this research identifies areas of high priority and a lack of studies about teacher training. This study provides potential future directions for medical education research for residents in radiation oncology.

## Background

In recent decades, both medical education and radiation oncology have achieved significant research progress. The numbers of publications, journals, and conferences related to medical education have increased substantially [1]. Meanwhile, with advancements in biology, clinical oncology, and technology, radiation oncology has been propelled to a higher level, and these advancements not only lead to improved treatment outcomes but also exert a major influence on economic considerations and education [2–4]. Advances in medical education in radiation oncology are anticipated because these two fields are evolving. A literature review regarding undergraduate medical education in radiation oncology noted the necessity for medical students to follow effective curricula [5]. However, to the best of our knowledge, no study has noted the aspects missing from medical education research for residents; we aim to fill this gap.

A generalized overview is necessary to understand the trends and identify the deficiencies in recent studies. Therefore, a scoping review was utilized in this study. In contrast to a systematic review that explores a specific question, a scoping review is designed to map the literature in certain areas. This mapping illustrates key concepts, main sources, and types of evidence. A scoping review is thus particularly useful for complex topics that cover various existing study designs but have not been extensively reviewed [6].

This study reveals recent research trends in medical education specifically for residents in radiation oncology through a scoping review. Topics about entering, during, and finishing radiation oncology resident physicians training program are included in this study. It also addresses some topics that are neglected in recent studies. Moreover, our findings provide direction for future medical education research in radiation oncology for residents.

## Methods

The methodological approach used in this scoping review was proposed by Arksey and O’Malley [6] and modified by Levac and Colquhoun [7]. A scoping review involves five stages: (1) Development of a research question: clarifying the purpose of the study; (2) Search of relevant studies: balancing feasibility with the breadth and comprehensiveness of the scoping process; (3) Selection of studies: using an iterative approach to select studies; (4) Data coding: extracting and charting data; and (5) Summary of results: incorporating a numerical summary and qualitative thematic analysis, reporting results, and considering the implications of the study findings with respect to policy, practice, or research [7].

### Stage 1: Development of research question

The research questions identified by the research team are as follows:

1. *What research areas have been investigated in postgraduate medical education research in radiation oncology for residents?*
2. *What topics are awaiting further investigation?*

### Stage 2: Search relevant studies

A 10-year period from 2008 to 2017 was selected to survey for the recent trends in the research of medical education for residents in radiation oncology. We searched through the following three online databases: MEDLINE, PubMed, and Scopus. We used the following search terms to identify relevant topics: radiation oncology, radiotherapy, or cancer. By means of the term “AND”, we combined these three terms with other terms: medical education, curriculum, learning, teaching, mentor, postgraduate, or resident. These terms were used in searching the titles, abstracts, keywords and MeSH terms of articles. Matched articles were managed using EndNote (version 7, Clarivate Analytics, Philadelphia, United States).

### Stage 3: Selection of studies

We included only studies whose complete article is available in English. We then narrowed the articles to those focusing on postgraduate medical education for radiation oncology residents. Moreover, the works we selected have quantitative, qualitative, or mixed method designs. We excluded articles focusing on scientific research (e.g. investigations about cancer treatment, diagnosis, radiobiology, or medical physics), patient education, and medical education for other trainees (e.g. undergraduate students, attending physicians, and resident physicians in specialties other than radiation oncology) or other medical professions (e.g. medical physicists, nurses, and technicians) as these articles are beyond the scope of this study. We also excluded commentary articles, letters, editorial articles, and papers focusing on curriculum development because of their lack of a research component. As suggested by Levac and Colquhoun, the study selection stage was an iterative process. Challenges and uncertainties related to study selection were identified during the article review. The search strategy was modified accordingly and discrepancies were resolved via discussion among our research team based on Levac and Colquhoun’s approach [7].

### Stage 4: Data coding

We first developed a data abstraction form. The initial backbone of the abstraction form was developed by including descriptive contents such as study characteristics, topic areas, and methodologies. A detailed revision was then performed by reading through a random selection of five articles, and the procedure was modified iteratively for final presentation of the data. Members of the research team read the included articles and extracted data into the final abstraction form. Uncertainties about abstraction were marked and later reviewed again. The research quality was not appraised because this scoping review aimed to identify the trends and themes of the targeted area to aid development in future research.

### Stage 5: Summary of results

For the final stage, we gathered the data from the abstraction form. The coded data were analyzed using Microsoft Excel 2010 (Microsoft, Redmond, United States) and summarized by a descriptive summary analysis. The results are presented in figures and tables and in narrative form to illustrate recent trends and patterns in the research on postgraduate medical education in radiation oncology for residents.

## Results

**Fig. 1:**
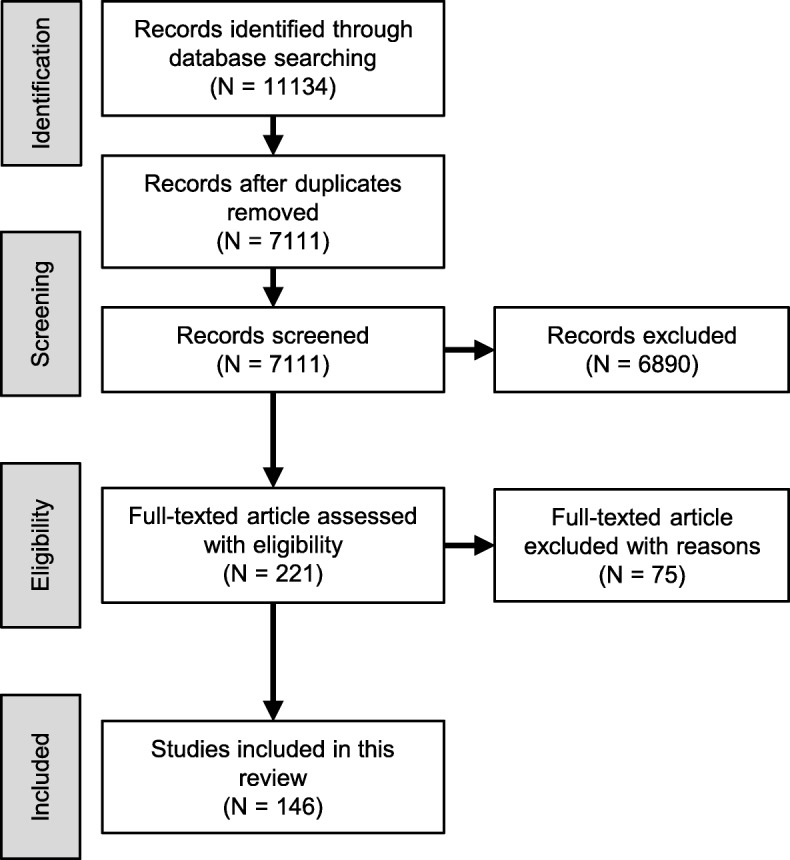
Preferred Reporting Items for Systematic Reviews and Meta-Analyses (PRISMA) flowchart of search results

We initially retrieved 11,134 citations from the MEDLINE, PubMed, and Scopus databases. After removing duplicates, a total of 7111 citations remained. We then excluded 6890 citations after screening the titles and abstracts on the basis of the inclusion and exclusion criteria. After screening the remaining 221 full-text articles, we further discarded 77 articles because they were articles of historical descriptions, conference papers, or only had abstracts written in English. Finally, 146 articles that met the inclusion criteria remained for data analysis. Our screening procedure is summarized in Fig. 1.

**Fig. 2:**
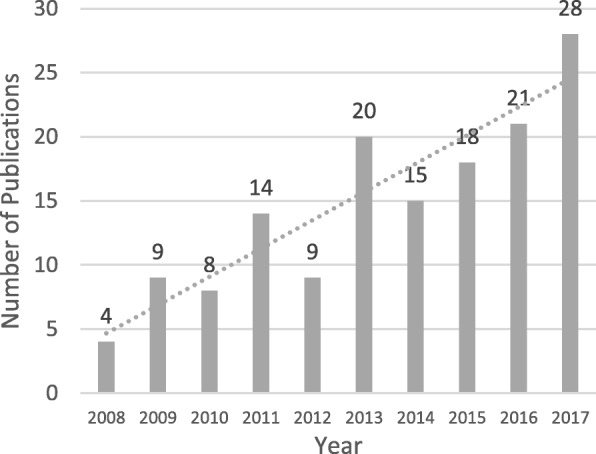
Number of publications per year

The included publications originated from 14 countries. The majority are from North America (103, 70.5%), and the remainder, which accounted for less than 30% of the studies, were from Europe (23, 15.8%), Oceania (14, 9.6%), Asia (5, 3.4%), and Africa (1, 0.7%). The following six countries with five or more articles are presented in Table 1: the United States (78, 53.4%), Canada (25, 17.1%), Australia (14, 9.6%), Denmark (5, 3.4%), France (5, 3.4%), and the United Kingdom (UK) (5, 3.4%). A mild increase was observed in the number of annual publications from 2008 to 2017 (Fig. 2).

**Table 1:** Study characteristics

Geographic distribution	No. of publications (%)
North America (USA., Canada)	103 (70.5)
Europe (Belgium, Denmark, France, Italy, Lithuania, UK)	23 (15.8)
Oceania (Australia)	14 (9.6)
Asia (India, Japan)	5 (3.4)
Africa (Nigeria)	1 (0.7)
Country (5 or more publications)	
United State of America	78 (53.4)
Canada	25 (17.1)
Australia	14 (9.6)
Denmark	5 (3.4)
France	5 (3.4)
United Kingdom	5 (3.4)
Study Methodology	
Quantitative	114 (78.1)
Qualitative	22 (15.1)
Mixed-Method	10 (6.8)

*Abbreviations*: *USA* United States of America, *UK* United Kingdom

**Fig. 3:**
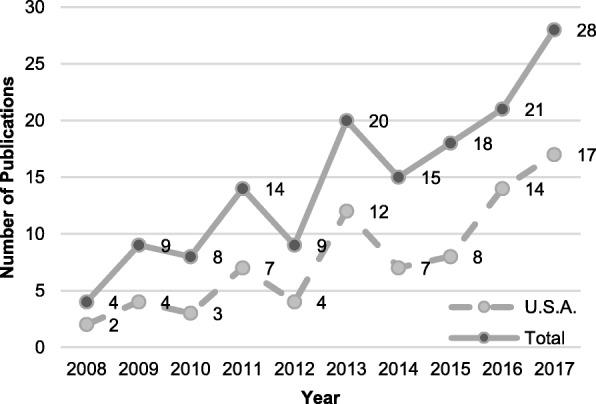
The line of total publication number per year and the publication number from the United States

A similar increase in the number of publications was observed when the scope was confined to the United States (Fig. 3). Regarding the methodologies, quantitative studies constituted the majority (114, 78.1%) and only 22 studies used qualitative methodologies. Meanwhile, fewer than 10% of the studies adopted a mixed-methods approach (10, 6.8%). A detailed distribution of the study methods is shown in Table 2. The most commonly used method among the included studies was primary methods of data collection (i.e., questionnaires, scoring evaluations, pre-test and post-test comparisons, and randomized trials), accounting for 78.1% (114). Documentation was used (i.e., exam/test result, publication search, and electronic record) in 21.9% (32) of the studies, followed by focus groups (6, 4.1%) and interviews (2, 1.4%).

**Table 2:** Distribution of study methods

Study methods	No. of publications (%)
Primary data collection	114 (78.1)
Questionnaires	97 (66.4)
Scoring evaluations	17 (11.6)
Pre-test and post-test comparisons	12 (8.2)
Randomized trials	5 (3.4)
Documentation	32 (21.9)
Exam/test results	2 (1.4)
Publication search	17 (11.6)
Electronic records	15 (10.3)
Focus groups	6 (4.1)
Interviews	2 (1.4)

**Fig. 4:**
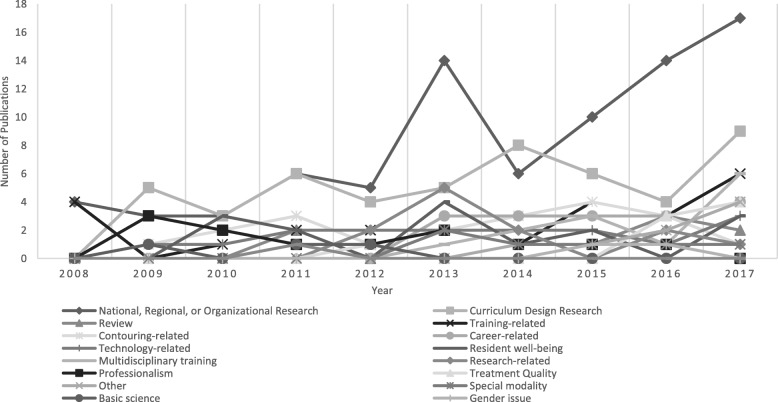
Trend of topics

The respective numbers of publications in the identified topic areas are presented in Table 3. Most of the publications fell into the categories of national, regional, or organizational research (82, 56.1%); that is, most studies were conducted to survey certain issues in an organization, a specific region, or a country. This category was followed by curriculum design research (50, 34.2%); and reviews (8, 5.5%). Other research topics with more than ten publications between 2008 and 2017 included: training (25, 17.1%), contouring (20, 13.7%), career (17, 11.6%), technology (16, 11.0%), resident well-being (15, 10.3%), multidisciplinary training (14, 9.6%), research (13, 8.9%), and professionalism (12, 8.2%). The numbers of publications inside and outside the United States are also presented in Table 3. When we compared the publication percentage in each topic, there were more publications related to national, regional, or organizational settings; training; career; resident well-being; and research in the United States. On the other hand, more studies regarding curriculum design, contouring, multidisciplinary training, professionalism, and technology-related topics were conducted in other countries. The trends of the topics are shown in Fig. 4, and the detailed information and numbers are presented in Additional file 1: Table S1. The increase in national, regional, or organizational research was in accordance with the increase in total publications. Training-, contouring-, and technology-related studies were persistently conducted during this period. Moreover, career-related issues were increasingly considered in the past 5 years. Moreover, increases in publications related to treatment quality and multidisciplinary training were observed in 2016 and 2017. Additionally, no study related to teacher training or resident-as-teacher was identified.

**Table 3:** Distribution of the included publications by category, and the number of publications in the United States of America (USA), other countries (Others), and their total

Topic area (detailed subjects)	No. of publications (%)		
	USA	Others	Total
National, Regional, or Organizational Research	52 (66.7)	30 (44.1)	82 (56.2)
Curriculum Design Research	20 (25.6)	29 (42.6)	50 (34.2)
Training-related (training content, needs, environment, examination, quality, fellowship, and mentorship)	15 (19.2)	10 (14.7)	25 (17.1)
Contouring-related (contouring, anatomy, and imaging)	8 (10.3)	12 (17.6)	20 (13.7)
Career-related (resident matching, career choice, and employment)	12 (15.4)	3 (4.4)	17 (11.6)
Technology-related (e-learning, mobile technology, online learning, online tool, and virtual environment)	6 (7.7)	10 (14.7)	16 (11.0)
Resident well-being (stress, finance, workplace bullying, parenthood, and policy)	11 (14.1)	4 (5.9)	15 (10.3)
Multidisciplinary training (interprofessional, internationals, and CME credit)	5 (6.4)	9 (13.2)	14 (9.6)
Research-related (research training, productivity, and trend)	10 (12.8)	3 (4.4)	13 (8.9)
Professionalism (communication skills, ethics, and clinical reasoning)	4 (5.1)	8 (11.7)	12 (8.2)
Treatment quality (safety and quality, and error-disclosure)	5 (6.4)	3 (4.4)	8 (5.5)
Supportive care (palliative treatment, cancer survivorship, and geriatric oncology)	6 (7.7)	2 (2.9)	8 (5.5)
Review	2 (2.6)	5 (7.2)	8 (5.5)
Special modality (particle therapy, brachytherapy, and SRS)	4 (5.1)	2 (2.9)	6 (4.1)
Basic science (radiation biology and medical physics)	3 (3.8)	0	3 (2.1)
Gender issue (Gender and pregnancy)	2 (2.6)	0	2 (1.4)
Learning process	0	1 (1.5)	1 (0.7)
Teaching training (teacher training and resident-as-teacher)	0	0	0

## Discussion

To the best of our knowledge, this work is the first literature review on medical education for residents in radiation oncology. We identified 146 articles published between 2008 and 2017. The trend of increasing publications and a relatively low number of publications in these years indicates the growing potential of the field. This finding strengthens related studies and provides a foundation for further radiation oncology medical education for residents.

Resources available for conducting research are limited. Therefore, a research stakeholder would choose the research topic matching their greater interest [8]. The trends of topics indicate that most stakeholders were interested in the overall picture of the current situation; therefore, the national, regional, or organizational research category accounted for the majority of research, and the number of related publications increased. Meanwhile, the importance of training, contouring, and technology was not neglected. Topics related to these three domains were consistently studied each year.

The largest number of publications was from the United States, followed by Canada and Australia. Two potential explanation exist for this phenomenon: first, the inclusion criterion of publications is the English language (these three countries are all English-speaking countries); second, radiotherapy and related equipment are advanced and continuously being developed in these countries. By contrast, we identified only one article from the African region. This significant difference may have resulted from resource shortages in such developing countries. Despite the fact that cases of cancer and cancer-related deaths are continuously rising in Africa, African countries face considerable challenges with respect to equipment and human resources [9]. Under these circumstances, medical education research in these countries would be less of a concern and therefore underdeveloped.

The United States had the largest number of publications; hence, the trend of its number of publications and that of the total number of publications are similar (Fig. 3). According to our results, the number of publications surged after 2010; this phenomenon was partially driven by the estimated workforce shortage. Smith et al. expected a 10 times faster growth in radiotherapy demand than supply between 2010 and 2020 [10]. Their study revealed a similar situation for the medical oncologist workforce due to the aging of the general population [11]. The issue of training more qualified radiation oncologists thus surfaced. Yang et al. predicted that workforce shortages will persist in 2025 under the existing conditions of aging and a better-insured population, and warned about the limited ability to provide high-quality cancer care [12]. These warnings resulted in continued interest in training-related medical education research for residents. Such considerations also gave rise to concerns about career-related topics; an increase in publications on career-related topics since 2013 was observed. In our survey, career-related topics included resident matching, career choice, and employment. Initially, most studies focused on resident matching, but the interest gradually shifted to career choices and employment after residency. As the plan for workforce expansion persisted, understanding the status of resident recruitment and the influence of undergraduate medical education on career choice became especially important [13, 14]. However, the studied subject changed from postgraduate choice to post-training choice. Presently, there is an excess of radiation oncologists in the United States, but the number of residency slots has continued to expand [15]. Meanwhile, trainees gave more value to future job procurement because of the perception of a tightening job market [16]. In summary, the increasing interest in career-related topics originated from the motivation to understand where trainees came from and where they end up. These studies are necessary to help the moderators of training programs understand how to recruit appropriate candidates for radiation oncology and to determine future employment and unemployment issues for current trainees.

As the resident slot expansion continues, how to provide trainees with adequate training in quality care and safety for patients has emerged as a critical issue. We found an increase in publications related to treatment quality in 2016 and 2017. Only one publication in this category was identified before 2016. Therefore, awareness of treatment quality is considered to be an increasingly important topic for future research. To further provide better quality care to cancer patients, an understanding of the contribution of other disciplines and high-quality training are required [17]. Another surge in publications related to multidisciplinary training was noted in 2017. In addition, multidisciplinary training in radiation oncology also includes medical physics [18]. The included studies address the necessity of more comprehensive training in both “radiation” and “oncology”.

Within this scoping review, we revealed a missing component in the research topics compared with medical education reviews or studies in other specialties [19–23]. Specifically, the research on teacher training in radiation oncology is insufficient. Although curriculum design research accounted for approximately one-third of the total publications in this study, teaching faculty training remains unexplored. Meanwhile, no studies on training residents as teachers were identified in the examined papers. The idea of the resident-as-teacher began around the 1960s and has become more common since the 1990s. Studies have demonstrated that one-third to two-thirds of undergraduate clinical medical education was provided by junior medical staff who spent 25% of their working week teaching [20]. Specialties such as radiology, surgery, gynecology, and emergency medicine have been developing resident-as-teacher training programs for years [19, 21, 23, 24]. Moreover, teaching is an essential competency for residents to achieve, and studies have revealed that residents’ teaching skills can and need to be improved [20, 23]. Implementation of a training program for teaching skills is necessary to develop the competency of residents and enhance the quality of education for undergraduates in radiation oncology.

The distributions of topic areas differ between the United States and other countries. Studies in the United States focused more on national, regional, or organizational research, in addition to career-related, research-related, and training-related content. An emphasis on these areas of interest reflects the issue of workforce adjustment during the past decade [12–15]. For countries other than the United States, more studies focused on curriculum design, contouring, multidisciplinary training, professionalism, and technology. Some of the studies aimed to catch up with technological development and to develop new tools [25–28]. Others explored the benefits of modern technology for balancing inadequate training due to local resource shortages [29]. The number of publications regarding working force or career-related issues was low in countries other than the United States. The lack of such relevant studies might imply either a balanced supply and demand in these regions or that the issue was known but ignored. Either way, the career-related issues should be investigated to determine the actual global workforce situation and to generate a solution to any existing or future crisis in the workforce via international collaborations. Moreover, the overall number of publications conducted was low in Asian countries. Aside from the potential language barrier, this observation highlights the relatively little interest in medical education research in Asia. The development of medical education in Asia was reported to be a low-priority research interest due to the known constraints of low socioeconomic conditions, cultural and religious values, lack of congruence, leadership crisis, lack of financial resources, inadequate training for medical education research, and unforeseeable outcomes [30]. The number of publications in Asia was negligible in a study of the geographic distribution of medical education almost two decades ago [31]. Nevertheless, increased academic productivity in medical education has been identified recently with the increasing global awareness of the importance of medical education [32]. The low number of Asian publications in this study indicates a delayed response to the overall academic productivity in Asia. Meanwhile, the proportion of medical education research conducted in Asia was 17% in the field of Neurosurgery. As a result, the language barrier should not be considered as a primary cause of low productivity shown in our study. Therefore, further medical education research in radiation oncology for residents should be promoted specifically in Asia.

In addition to the comparisons within our study, some differences between medical education research in radiation oncology and in family medicine were noted [22]. Aside from the professional differences among specialties, the results for family medicine showed a much lower interest in resident well-being (only 1 article, less than 1%) than indicated by our results (15 articles, 10.2%). Topics regarding training for teachers accounted for 4% (25 articles) of the studies on family medicine, whereas we found no literature on this topic within our scope. Both studies described a consistent finding of quantitative methodology as the leading approach, and the most commonly used methods were primary methods of data collection and documentation. This similarity is attributed to the benefits of Internet use which enables easy access to questionnaires via e-mail or online surveys. Meanwhile, publication search and electronic records are currently much easier to achieve through the Internet than in the past times when only hard copies of publications or records were available.

A limitation of this study is that only English publications were included. Seven publications were excluded because of the language criterion. The number of excluded publications would affect the final result of the regional distribution of the included studies, thus neglecting the potential influences of studies from non-English speaking countries. Furthermore, the quality of publications was not assessed. Given the nature of a scoping review, detailed information about a particular thematic area is usually not required. Future reviews that consider specific topics within the field of radiation oncology education research in depth are highly recommended.

## Conclusion

This study is the first scoping review on medical education regarding residents and radiation oncology. A total of 146 articles in a 10-year period might not appear to be a significant number; however, the findings of this study highlight the trend in the interest of the stakeholders of medical education research in radiation oncology. While topics such as training-, contouring-, and technology-related issues were persistently valued, career-related, multidisciplinary training, and treatment quality studies are receiving increasing interest. These studies showed that issues related to the workforce and training qualified physicians remained core values for researchers. However, the importance of teacher training was neglected; therefore, more attention is required to improve teaching quality and residents’ competency as teachers. For future research in regions other than the United States, studies on issues related to career and workforce should be encouraged. Last, it is essential to promote additional relevant studies in Asian countries to determine Asian educators’ perspectives toward medical education research in radiation oncology for residents.

## Supplementary information

**Additional file 1 **: **Table S1.** Distribution of the included publications by the categories and years.

## Data Availability

The datasets used and/or analyzed the current study are available from the corresponding author upon reasonable request.

## Abbreviations

UK: United Kingdom
USA: United States of America
