# Supplementary material for: A scoping review of medical education research for residents in radiation oncology
Source: BMC Med Educ. 2020 Jan 13;20:13. doi: 10.1186/s12909-020-1927-x (PMC6958614; doi:10.1186/s12909-020-1927-x)
Supplement: Supplementary file 1 — Additional file 1 : Table S1. Distribution of the included publications by the categories and years. [file 12909_2020_1927_MOESM1_ESM.docx]

**Additional file**

Tabel S1. Distribution of the included publications by the categories and years.

| Topic Year | 2008 | 2009 | 2010 | 2011 | 2012 | 2013 | 2014 | 2015 | 2016 | 2017 | Total no. |
| --- | --- | --- | --- | --- | --- | --- | --- | --- | --- | --- | --- |
| National, Regional, or Organizational Research | 4 | 3 | 3 | 6 | 5 | 14 | 6 | 10 | 14 | 17 | 82 |
| Curriculum Design Research | 0 | 5 | 3 | 6 | 4 | 5 | 8 | 6 | 4 | 9 | 50 |
| Review | 0 | 0 | 0 | 2 | 0 | 0 | 0 | 1 | 3 | 2 | 8 |
| Training-related | 4 | 0 | 1 | 2 | 2 | 2 | 1 | 4 | 3 | 6 | 25 |
| Contouring-related | 0 | 1 | 2 | 3 | 1 | 2 | 3 | 4 | 3 | 1 | 20 |
| Career-related | 0 | 0 | 0 | 1 | 0 | 3 | 3 | 3 | 3 | 4 | 17 |
| Technology-related | 0 | 1 | 1 | 2 | 2 | 2 | 2 | 2 | 1 | 3 | 16 |
| Resident well-being | 0 | 0 | 3 | 2 | 0 | 4 | 1 | 2 | 0 | 3 | 15 |
| Multidisciplinary training | 0 | 0 | 0 | 1 | 0 | 1 | 2 | 3 | 1 | 6 | 14 |
| Research-related | 0 | 1 | 0 | 0 | 2 | 5 | 2 | 0 | 2 | 1 | 13 |
| Professionalism | 0 | 3 | 2 | 1 | 1 | 2 | 1 | 1 | 1 | 0 | 12 |
| Treatment Quality | 0 | 0 | 0 | 0 | 1 | 0 | 0 | 0 | 3 | 4 | 8 |
| Other | 0 | 0 | 0 | 0 | 0 | 0 | 1 | 1 | 2 | 4 | 8 |
| Special modality | 0 | 0 | 0 | 0 | 0 | 2 | 1 | 1 | 1 | 1 | 6 |
| Basic science | 0 | 1 | 0 | 1 | 1 | 0 | 0 | 0 | 0 | 0 | 3 |
| Gender issue | 0 | 0 | 0 | 0 | 0 | 0 | 0 | 1 | 1 | 0 | 2 |
| Learning process | 0 | 0 | 0 | 1 | 0 | 0 | 0 | 0 | 0 | 0 | 1 |
| Teaching training | 0 | 0 | 0 | 0 | 0 | 0 | 0 | 0 | 0 | 0 | 0 |
